# Vitamin D levels in the pre- and post-COVID-19 pandemic periods in pediatric patients with chronic kidney disease

**DOI:** 10.3389/fnut.2023.1268347

**Published:** 2023-11-03

**Authors:** Israel Parra-Ortega, Jessie Nallely Zurita-Cruz, Itzel Ortiz-Flores, Benjamin Romero-Navarro, Miguel Angel Villasis-Keever, Briceida López Martínez, Veronica Domínguez-Castillo, José Carlos Romo-Vázquez

**Affiliations:** ^1^Auxiliary Diagnostic Services, Hospital Infantil de México Federico Gómez, Ministry of Health (SSA), Mexico City, Mexico; ^2^Facultad de Medicina Universidad Nacional Autónoma de México, Hospital Infantil de México Federico Gómez, Mexico City, Mexico; ^3^Analysis and Synthesis of the Evidence Research Unit, National Medical Center XXI Century, Instituto Mexicano del Seguro Social, Mexico City, Mexico; ^4^Department of Teaching and Research, Laboratorios Ruiz SYNLAB, Mexico City, Mexico; ^5^Department of Pediatric Nephology, Hospital Infantil de México Federico Gómez, Ministry of Health (SSA), Mexico City, Mexico

**Keywords:** COVID-19 pandemic, vitamin D, chronic kidney disease, pediatric, cholecaciferol

## Abstract

**Introduction:**

Vitamin D (VD) deficiency is common in children with chronic kidney disease (CKD) because of multiple factors. During the coronavirus disease 2019 (COVID-19) pandemic, it increased because of medicine shortage and no enough medical service for patients with non-COVID-19 diseases.

**Objective:**

To analyze the effects of the COVID-19 pandemic-related lockdown on the serum levels and status of 25-hydroxyvitamin D3 (25-[OH]D) in children with CKD.

**Materials and methods:**

This retrospective study included patients (6–18 years old) who were diagnosed with CKD stage 2–5 and routinely measured for serum VD levels between May 2019 and December 2022. Serum 25-(OH)D levels were measured before, during, and after the pandemic (2019, 2020–2021, and 2022, respectively). The daily dose of cholecalciferol supplementation and the readjustment (if required) were recorded.

**Results:**

This study included 171 patients (median age: 12 years). Before the pandemic, the median serum VD level was 25.0 ng/mL (19.3% VD deficiency). Then, VD supplementation was adjusted to 400–1,200 UI daily in 98.8% (*n* = 169) of patients. During the pandemic, the median VD level decreased to 22.5 ng/mL (43.3% VD deficiency). Hence, the supplementation was readjusted, and after the pandemic, the level was 28.7 ng/mL (18.7% VD deficiency), indicating a statistically significant increase in serum VD levels from the prepandemic period (*p* = 0.007).

**Conclusion:**

Decreased serum VD levels and increased VD deficiency frequency were observed in patients with CKD during the COVID-19 but improved after readjustment of supplementation.

## 1. Introduction

Vitamin D (VD) is a fat-soluble steroid hormone that has a specific cytosolic receptor. Initially, VD was related to calcium and phosphorus metabolism; however, more recently, it has been found to have a role on multiple central extraskeletal effects on several target organs, such as adipose tissue, blood cells, the immune system, skin, muscles, endocrine pancreas, and blood vessels (1, 2). The VD receptor (VDR), which is expressed in almost all organs, acts via the genomic (nuclear VDR) and nongenomic (membrane VDR) pathways. Humans acquire most of their VD from sunlight-induced cutaneous synthesis (approximately 80%), and the remainder from diet and supplementation (3, 4). However, the factors associated with VD deficiency included dark skin, sedentary periods, insufficient sun exposure, air pollution, obesity, and lack of VD supplementation (5).

VD is also important in chronic kidney disease (CKD)-related mineral bone disorder, considering that 1-α hydroxylase, which is essential for bone formation and resorption, is found in the kidneys. Low serum levels of 25-hydroxyvitamin D3 (25-[OH]D) cause negative calcium balance, secondary hyperparathyroidism, and bone disease. In CKD, hyperphosphaturic osteocyte-derived hormone (FGF-23) increases to compensate for phosphate retention and further inhibits renal 1α-hydroxylase expression and induces 24-hydroxylase expression responsible for 1,25(OH)D degradation. Poor 25(OH)D absorption caused by kidney disease is the main cause of 1,25(OH)D deficiency (4). Thus, patients with terminal CKD are deficient in activated VD as well as nutritional VD (6).

Taking into account that serum 25(OH)D level <20 ng/mL indicates VD deficiency, and >30 ng/mL is needed for optimal health, the Kidney Disease Outcomes Quality Initiative (KDOQI) and Kidney Disease Improving Global Outcomes (KDIGO) guidelines recommend measuring 25(OH)D levels once a year in children with CKD stages 2–5 and starting supplementation if levels are <30 ng/mL (7). The KDOQI guidelines also recommend the administration of cholecalciferol for treating VD failure in CKD stages 3 and 4, and active VD hormone for VD deficiency in patients with stage 5 CKD who also have secondary hyperparathyroidism. The data clearly indicate that 25(OH)D insufficiency persists as patients progress from stage 3 to stage 5 CKD (8).

During the coronavirus disease 2019 (COVID-19) pandemic, people were quarantined for 1 year (2020) (9). In Mexico, children were quarantined for 2 years (2020–2021), predisposing them to possible significant long-term effects on health. Confining oneself indoors for a longer period reduces sunlight exposure time, leading to a decrease in cholecalciferol synthesis. On the other hand, during the pandemic, there were medicine shortages and patient care was limited, including those with chronic diseases. Epidemiological studies have reported that the COVID-19 pandemic has changed the VD levels in children (10, 11). Patients suffering from chronic diseases, such as CKD, are highly at risk for VD deficiency (12), which might have worsened during the pandemic; however, data from the COVID-19 pandemic period are lacking or conflicting.

Hence, our study aimed to analyze the effects of the COVID-19 pandemic-related lockdown on the serum levels and status of 25-(OH)D in pediatric patients with CKD.

## 2. Materials and methods

### 2.1. Subjects

In Mexico, the first case of COVID-19 was reported on February 27, 2020, and a few days later (March 11, 2020), the WHO officially declared a pandemic. On March 31, 2020, the Mexican government officially announced a nationwide lockdown, which implies the total closure of schools, universities, public squares, and all shops, except for supermarkets, grocery stores, and pharmacies (13, 14).

This is a retrospective cohort study with pediatric patients with CKD stages 2–5 who, aged 6–18 years old. All patients were routinely measured for serum VD levels before and after the pandemic period at a tertiary pediatric hospital (Hospital Infantil de Mexico Federico Gómez) in Mexico City between May 2, 2019 and December 31, 2022. All patients were treated in the outpatient clinic of the Nephrology department, and met the definition of CKD proposed by KDIGO, since they had deterioration in renal function >3 months, and to determine the severity of CKD each patient was staged according to the KDIGO criteria (stages 2 to 5). Patients with diseases that prevent normal metabolism of the VD were excluded (diabetes, heart disease, or had been treated for cancer within the previous 5 years or genetic rickets), as well as those with incomplete clinical and biochemical evaluation. The cohort follow-up duration was 36 months. All included patients were selected using a consecutive sampling technique.

As of 2019, the hospital laboratory began to measure 25(OH)D because it was previously requested externally. In other words, not all patients underwent 25(OH)D measurement. Hence, the demographic and anthropometric data were collected at the beginning of the follow-up, particularly from the nephrology consultation in 2019 (pre-COVID-19 pandemic). We collected the data on the serum concentrations of 25(OH)D, the dose of cholecalciferol supplementation, and the need for supplementation readjustment from that consultation. After 24 months follow-up and COVID-19 immunization among adults in Mexico, the attendance of consultation in patients with CKD was regularized in 2021. From this consultation and 6–12 months thereafter, the data on 25(OH)D levels, the cholecalciferol supplementation dose, and the need for another supplementation readjustment were collected. At follow-up, adherence to VD supplementation was assessed.

### 2.2. Variables

The main outcome measures were serum 25(OH)VD concentrations and VD status, and the primary exposure variable was the cholecalciferol supplementation doses administered during the 36 months of surveillance. Serum 25(OH)VD concentrations and VD status at the beginning of follow-up were considered as predictors; while adherence to treatment, the COVID-19 pandemic period, and whether the patient had a kidney transplant were the confounding variables.

Patients with body mass index (BMI) <5th percentile were considered malnutrition, normal with BMI >5th percentile and <85th percentile, obesity with BMI > 95th percentile, and overweight with BMI > 85th percentile, according to the 2000 CDC Growth Charts (15). Patients with <2 standard deviations of height for age, BMI was calculated considering the age that corresponds to the 50th percentile of actual height.

**Figure 1:**
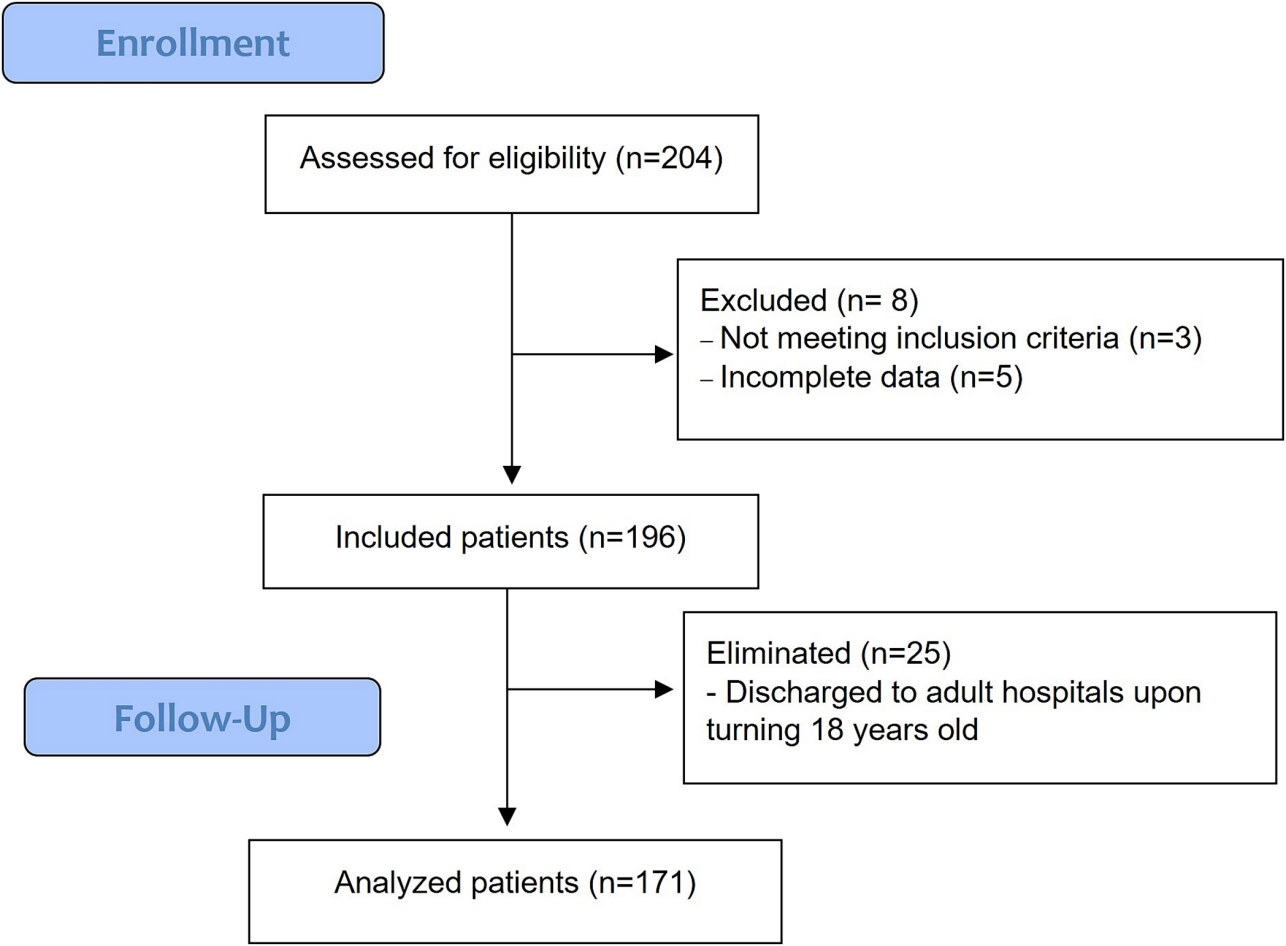
Flow diagram of individuals at each stage of study.

From 204 potentially eligible patients, three did not meet the inclusion criteria (two cancer patients undergoing chemotherapy treatment, and one patient with tetralogy of Fallot), and another five patients were excluded because of incomplete medical records. 25 patients were eliminated because they were discharged to adult hospitals upon turning 18 years old. Thus, 171 patients were analyzed (Figure 1).

According to the Declaration of Helsinki, the study protocol was approved by hospital ethics and research committee (registry number: HIM-2020-023).

### 2.3. Vitamin D determination

In the Hospital Infantil de Mexico Federico Gomez clinical laboratory, the serum concentrations of 25(OH)D were measured using the Abbot chemoluminescence technique with the equipment Archirech 1,000. A serum level of <20 ng/mL was considered as VD deficiency, 20–29.99 ng/mL as insufficiency, and >30 ng/mL as normal (12).

### 2.4. Statistical analyses

The quantitative variables were analyzed using Shapiro–Wilk test, and a nonparametric distribution was observed. We calculated the median and interquartile range (IQR) for the quantitative variables and the frequency and percentage for the qualitative variables. Differences in 25(OH)D at study onset, after 24 months, and 30–36 months were determined using the repeated measures Wilcoxon test. We also used Mann–Whitney *U* test and chi-square test for group comparison.

A value of *p* <0.05 was considered statistically significant. STATA v.12.0 (Stata Corp., College Station, TX, United States) was used for all statistical analyses.

## 3. Results

### 3.1. Baseline

Table 1 shows the baseline characteristics of the 171 included patients, who were 9–14 years old and had similar sex ratio. The majority had a normal nutritional status (68.4%), while 32 (18.7%) were overweight or obese. During evaluation, 110 (64.3%) had stage 2 CKD, followed by 35 (20.5%) with stage 5 CKD. Of these patients with stage 5 CKD, 26 (74.3%) underwent hemodialysis as replacement treatment, 7 (20.0%), and 2 underwent kidney transplant (Table 1).

**Table 1:** Baseline characteristics of pediatric patients with chronic kidney disease before the COVID-19 pandemic period (2019).

Characteristics	Total *n* = 171	
Age, *y*		
	Median (IQR)	12.0 (9.0, 14.0)
Sex, %		
	Female	93 (54.4)
	Male	78 (45.6)
Nutritional status, %		
	Normal	117 (68.4)
	Malnutrition	22 (12.9)
	Overweight/obesity	32 (18.7)
Stage chronic kidney disease, %		
	2	110 (64.3)
	3	12 (7.0)
	4	14 (8.2)
	5	35 (20.5)
CKD etiology		
	CAKUT	93 (54.4)
	Glomerulopathy	33 (19.3)
	Immunological	28 (16.4)
	Nephrectomy for neoplastic pathology	11 (9.9)
Cholecalciferol supplementation, %		
	Yes	81 (47.3)
Cholecalciferol supplementation dose, UI (*n* = 81)		
	Median (IQR)	400 (400, 800)
25(OH)D, ng/mL		
	Median (IQR)	25 (20.7, 29.8)
Vitamin D status, %		
	<20 (deficiency)	33 (19.3)
	20–29.9 (insufficiency)	98 (57.3)
	≥30 (normal)	40 (23.4)

25(OH)D, 25-hydroxyvitamin D3; IQR, interquartile range; CAKUT, congenital anomalies of the kidney and the urinary tract; CKD, chronic kidney disease.

**Figure 2:**
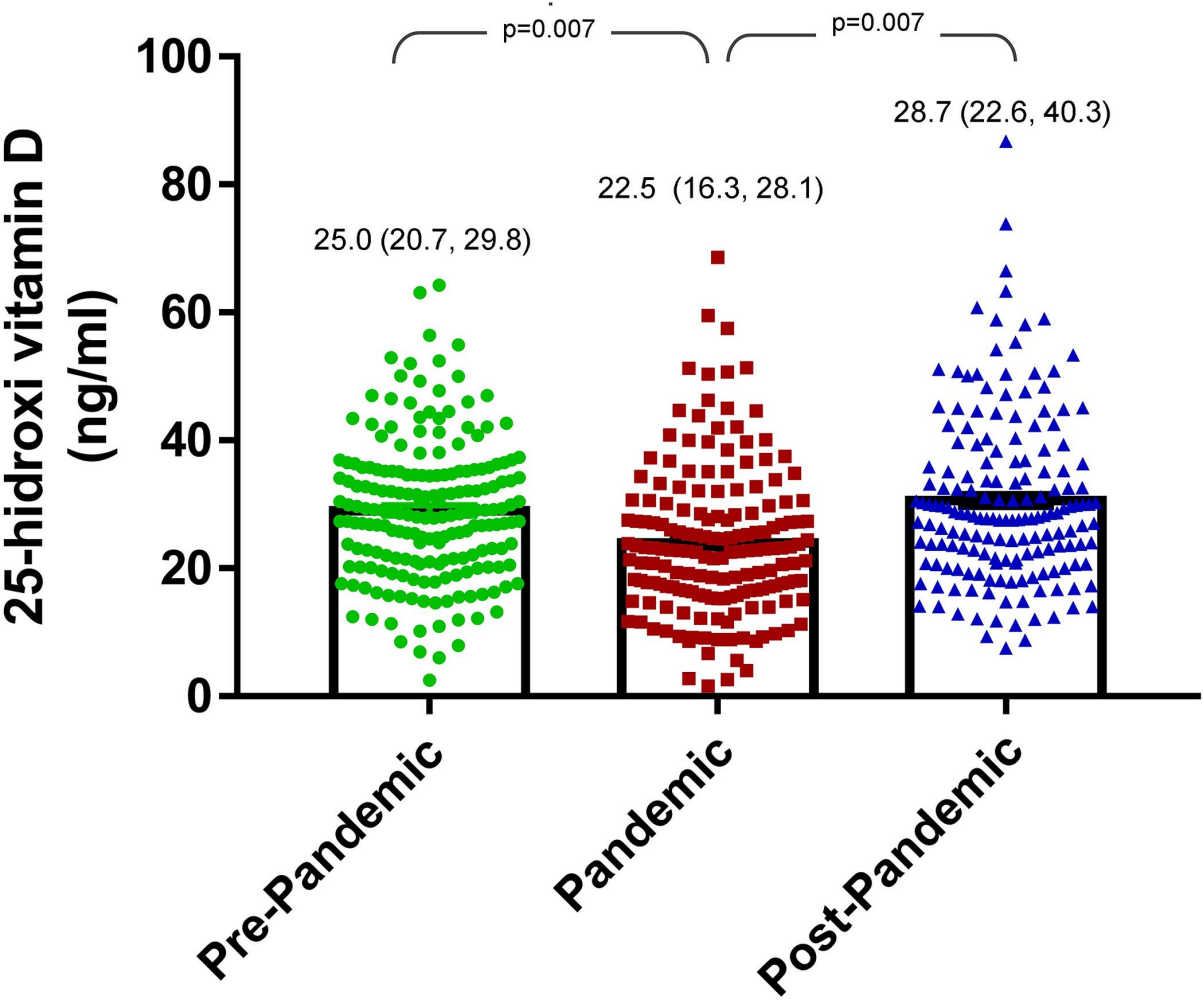
Change in vitamin D levels from before, during, and after the COVID-19 pandemic in pediatric patients with chronic kidney disease.

**Figure 3:**
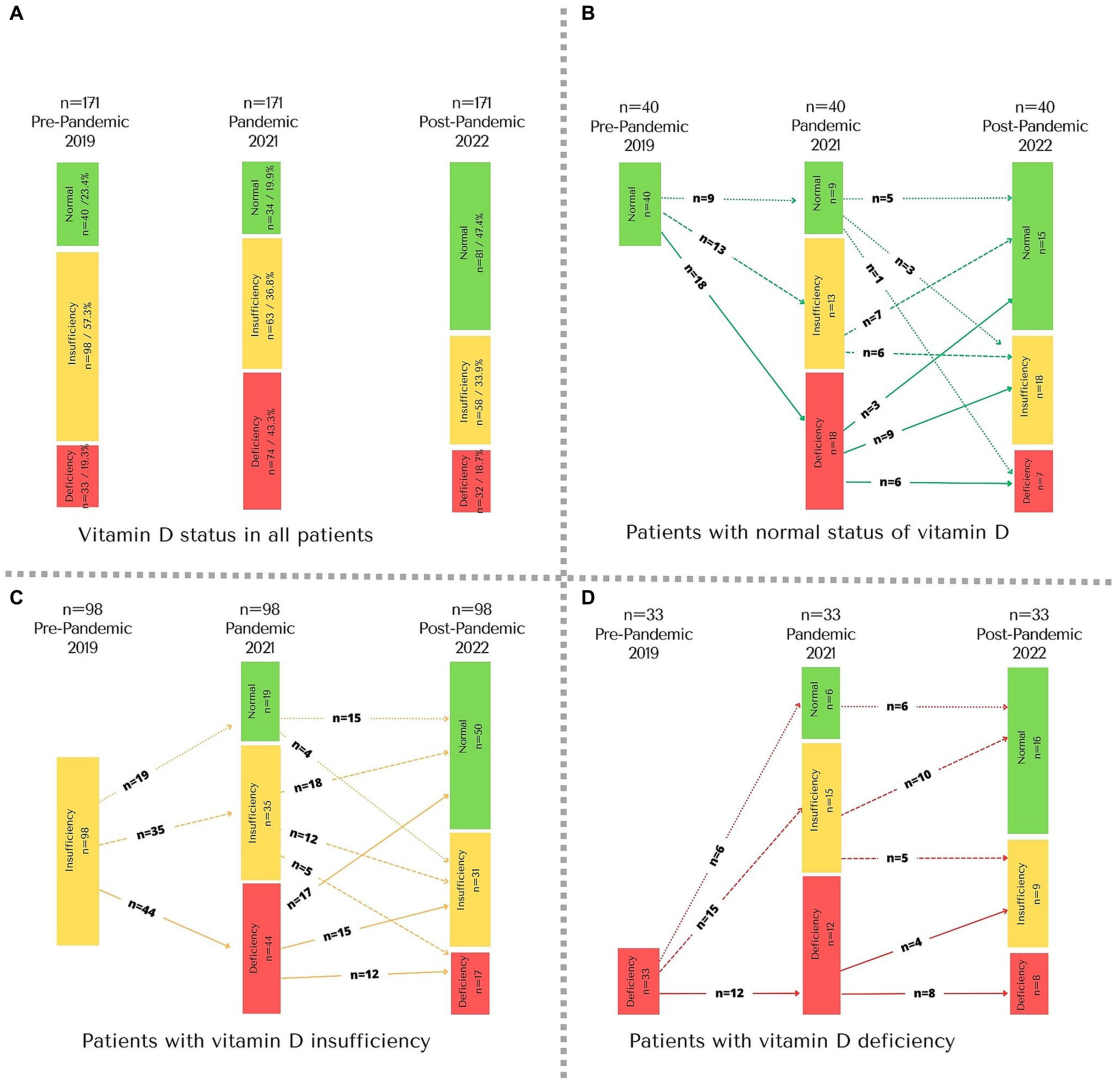
Change in vitamin D status from before, during, and after the COVID-19 pandemic in pediatric patients with chronic kidney disease. **(A)** All patients; **(B)** patients with normal status of vitamin D before the COVID-19; **(C)** patients with vitamin D insufficiency before the COVID-19; **(D)** patients with vitamin D deficiency before the COVID-19.

The median serum VD level among all patients was 25.0 ng/mL (IQR: 20.7, 29.8 ng/mL) (Table 1 and Figure 2); but only 23.4% (*n* = 40) had normal VD levels, 33 (19.3%) patients had VD deficiency and 98 insufficiency (57.3%) (Figure 3). As for supplementation, during this period 47.3% received cholecalciferol supplementation at a median dose of 400 IU per day (IQR: 400 to 800 IU). Therefore, according to VD serum levels, cholecalciferol supplementation was readjusted to a median of 800 IU per day (IQR: 400 to 1,200 IU) in 98.8% of the patients.

### 3.2. Follow-up (24 months)

After 24 months follow-up, the median 25(OH)D level was 22.5 ng/mL (IQR 16.3, 28.1 ng/mL) (Figure 2), and only 19.9% (*n* = 34) had normal levels, indicating a statistically significant decrease compared to baseline data (*p* = 0.007). The median delta 25(OH)D level from the baseline to the 24 months follow-up was −3.1 ng/mL (IQR: −11.9, 5.7 ng/mL).

Thus, the VD deficiency worsened as compared with that at the first evaluation (19.3% vs. 43.3%, *p* < 0.001) (Figure 3A). The 33 patients who had VD deficiency at baseline, 36.4% (*n* = 12) remained deficient and 45.5% (*n* = 15) progressed to insufficiency (Figure 3D). In the case of the 98 patients with VD insufficiency, 44.9% (*n* = 44) progressed to deficiency and 35.7% (*n* = 35) remained as insufficient (Figure 3C). Lastly, among the 40 patients with normal VD levels, most progressed to levels considered deficiency or insufficiency (*n* = 31, 77.5%) (Figure 3B).

During the pandemic, at 24 months of the follow-up, the median cholecalciferol supplementation was 800 IU per day (IQR, 400 to 1,200 IU per day). At 24 months of the follow-up was identified that up to 67.2% (*n* = 115) did not present adequate adherence to cholecalciferol supplementation.

### 3.3. Follow up (36 months)

After 6–12 months of follow-up of the cholecalciferol supplementation readjustment (30–36 months of follow-up at the beginning of the cohort study), the median serum VD level was 28.7 ng/mL (IQR: 22.6, 40.3 ng/mL) (Figure 2), where 47.4% (*n* = 81) had normal VD levels (Table 2). Thus, the level showed a statistically significant increase compared with the previous measurement (22.5 ng/mL vs. 28.7 ng/mL, *p* = 0.007) (Figure 2), as well as an increase in the proportion of patients with normal VD levels (19.9% vs. 47.4%) (Table 2). The median delta 25(OH)D level between the 24 months follow-up and the 30–36 months follow-up was 8.4 ng/mL (IQR: −0.5, 17.6 ng/mL).

**Table 2:** Characteristics of pediatric patients with chronic kidney disease after the pandemic period.

Characteristics		24 months after (2021)	30–36 months after (2022)	*p*
		Total *n* = 171		
Cholecalciferol supplementation, %				
	Yes	169 (98.8)	171 (100)	0.889
Cholecalciferol supplementation dose, UI				
	Median (IQR)	800 (400, 1,200)	4,000 (2000, 4,000)	<0.001
25 (OH)D, ng/mL				
	Median (IQR)	22.5 (16.3, 28.1)	28.7 (22.6, 40.3)	0.007
Vitamin D status, %				
	<20 (deficiency)	74 (43.3)	32 (18.7)	<0.001
	20–29.9 (insufficiency)	63 (36.8)	58 (33.9)	
	≥30 (normal)	34 (19.9)	81 (47.4)	

25(OH)D, 25-hydroxyvitamin D3; IQR, interquartile range.

During consultation and VD level verification, cholecalciferol supplementation was readjusted to a median dose of 4,000 IU per day (IQR, 2,000 to 4,000 IU; *n* = 78 [45%] dose ≤2,000 IU per day). Hence, cholecalciferol supplementation was increased as compared to that at the first evaluation (cholecalciferol supplementation 800 UI vs. 4,000 UI per day, *p* < 0.001). The patients were also recommended to increase intake of foods with a high VD content (Table 2).

At the end of follow-up (36 months), of the patients who started with VD deficiency, eight persisted with deficiency and 16 achieved normal levels (Figure 3D); Of the patients with insufficiency, 17 presented deficiency and 51.0% (*n* = 50) achieved normal levels (Figure 3C) and of the patients who started with VD sufficiency, 17.5% (*n* = 7) ended up with VD deficiency (Figure 3B).

At the end of follow-up (36 months), it was identified that 40 (23.4%) patients did not present adequate adherence to cholecalciferol supplementation, including the 32 patients who ended up with deficiency and 8 patients with VD insufficiency. It should be noted that of the 8 patients who persisted with deficiency during follow-up did not present adequate adherence to cholecalciferol supplementation. The median delta 25(OH)D level between the 24 months follow-up and the 30–36 months follow-up in patients did not present adequate adherence to cholecalciferol supplementation was lower that (0.59 ng/mL [IQR: −4.84, 7.6 ng/mL]) patients with adequate adherence (12.1 ng/mL [IQR: 3.29, 19.6 ng/mL]) (*p* < 0.0001).

### 3.4. Confounding variables

The proportion of patients with VD deficiency was lower at the end of the follow-up than at the beginning (18.7% vs. 23.4%, *p* = 0.04). This result is related to the increase in cholecalciferol dose compared with the dose at the initial follow-up (4,000 UI vs. 400 UI, *p* < 0.001).

10 patients underwent kidney transplantation, with no statistical difference in VD levels in the post-COVID-19 pandemic period between patients with and without kidney transplantation (26.4 ng/mL in those with kidney transplant vs. 21.8 ng/mL in those without kidney transplant; *p* = 0.225).

## 4. Discussion

VD deficiency and insufficiency have now become a global public health problem despite the availability of supplements. In pediatric patients with CKD, its prevalence has reached 62.5 to >80% (16, 17), similar to our study finding prior to the start of the pandemic (76.6%).

Different tissues unrelated to calcium and phosphorus metabolism express VDR and 1 alpha-hydroxylase (CYP27B1), which regulates gene expression in various tissues (18). Hence, VD is important in extraskeletal tissues as well as at the immunological, metabolic, and cardiovascular levels, among other systems (19, 20).

The most abundant source of cholecalciferol is from cutaneous synthesis on UV-B exposure from sunlight (21). Therefore, low sun exposure, sunscreen use, dark skin, and pollution lead to a decrease in VD synthesis (22). In addition, the dietary sources of cholecalciferol are limited to fatty fish, beef liver, and egg yolk, and Mexico has no fortified foods (23). Some chronic diseases, such as CKD, obesity, and diabetes mellitus, can also result in the decrease in VD bioavailability, worsening the VD levels (24).

During the COVID-19 pandemic, many factors contributed to VD level decrement; one factor was social confinement, which decreases sun exposure (10, 25). In Mexico, social confinement of children lasted for almost 2 years (2020 and part of 2021). Other factors include low intake of high VD food sources and shortage in medications, including cholecalciferol. In addition, contagion in Mexico City during the first year of the pandemic was positively related to COVID-19 mortality (26).

We found several retrospective studies that demonstrate the impact of restrictions during the COVID-19 pandemic on VD levels in the pediatric population, and VD insufficiency and deficiency were observed (25). Beyazgül et al. evaluated the impact of the first year of the pandemic on VD levels in preschoolers, schoolchildren, and adolescents (1–6, 6–12, and 12–18 years, respectively) at two different periods (pre- and post-pandemic); the found that the rate of deficiency values was significantly higher during the pandemic in schoolchildren and adolescents, with the latter group being the most affected (median VD level: 11.20 ng/mL, *p* = 0.003) (10). During the pandemic, our study similarly identified a statistically significant decrease of 3.1 ng/mL in serum VD levels in pediatric patients who received cholecalciferol supplements at a median dose of 800 IU per day. Therefore, we can assume that multiple factors negatively influenced these levels during the pandemic period, thereby increasing the need for closer monitoring and frequent supplementary readjustments. Similarly, Wong et al. conducted a retrospective study with a similar methodology wherein blood samples from 303 patients aged 2–24 months were analyzed; they found that patients recruited during the pandemic period had decreased VD levels (*p* < 0.001) (11). Dyussenova et al. also retrospectively examined 40 children with CKD with KDIGO stage 1–5 classification between January 2020 and September 2020 and demonstrated that 62.5% of these children had VD deficiency and that low levels correlate with a decrease in the glomerular filtration rate (17).

Populations vulnerable to VD deficiency are those with clinical conditions such as osteoporosis (primary or secondary), metabolic bone diseases, chronic renal disease, malabsorption syndrome, liver failure, type 1 diabetes mellitus, and cancer who could benefit from 25(OH)D concentrations maintained at 30–60 ng/mL (27).

The initial cholecalciferol supplementation in our pediatric population was only between 400 and 1,200 IU daily prior to the start of the pandemic, mainly because our country did not have pediatric presentations that had doses greater than 1,200 IU and children were also recommended to consume foods with high VD content to achieve adequate supplementation (2,000–4,000 IU), as recommended in patients with CKD (28). However, after the first year of the pandemic, new presentations of cholecalciferol from 2,000 to 4,000 IU emerged, as well as lower VD levels than those previously found in patients (25.0 ng/mL vs. 22.5 ng/mL, *p* = 0.007); the supplementation was then adjusted to a dose between 2,000 and 4,000 IU. With this latest adjustment, we observed that the proportion of VD deficiency decreased and that the VD levels were higher than those presented before the start of the pandemic (29).

In a healthy pediatric population, VD supplementation at a dose of 2,000 IU per day for 6 months to reach levels >30 ng/dL of 25[OH]D is effective up to approximately 92.9% (30). In the present study, after the pandemic, the dose of cholecalciferol in 45% of the patients was ≤2,000 IU per day, so most probably the majority of patients had low serum levels of VD. However, in studies in CKD pediatric patients with VD supplementation at doses of 4,000 IU, 36 to 91% achieved levels >30 ng/mL (31, 32); these data seem to correspond to our results, since despite cholecalciferol supplementation (up to 4,000 IU per day), at the end of follow-up only 47.4% reached VD sufficiency levels. The failure to achieve optimal VD levels, despite adequate supplementation, has been explained previously; Demburg et al. reported that glomerular disease may be associated with changes in vitamin D metabolism that is affected by urinary losses of vitamin D-binding protein (VDBP) secondary to glomerular and/or tubular damage in CKD (33). Furthermore, it has been observed a complex relationship between VDBP, free-25OHD and biologically available form of 25OHD in children with renal disease (33, 34). Therefore, it seems necessary to conduct pharmacokinetic studies of VD in CKD pediatric patients to better understand the relationship of dose supplementation and inter-individual variation.

The extraskeletal effects demonstrated that increasing serum 25(OH)D suggested promising effects in reducing the progression to diabetes mellitus type 2, decreasing cancer mortality, and reducing the incidence of autoimmune diseases (35–37).

In patients with CKD, nutritional supplementation with VD has potential benefits, including reduction of parathyroid hormone levels, beneficial impact on arterial and cardiac diseases, improvement of response to erythropoietin-stimulating agents, proteinuria decreases in respiratory and gastrointestinal infections of viral origin (38, 39).

One of the strengths of the study is that it is a longitudinal study with various measurements of VD levels, as well as modifications in VD supplementation with cholecalciferol. However, the index of sun exposure, food intake, or drugs that can modify VD bioavailability was not examined; therefore, these data are limited to a similar population according to the index of sun exposure and chronic disease. Furthermore, the observational nature of the study, while demonstrating associations, does not allow the drawing of definitive conclusions about causality.

In conclusion, decreased serum levels of VD and increased frequency of VD deficiency were observed during the COVID-19 pandemic in patients with CKD but improved after the readjustment of cholecalciferol supplementation. However, given that not all patients achieved normal VD levels, continuous monitoring seems necessary.

## Data availability statement

The raw data supporting the conclusions of this article will be made available by the authors, without undue reservation.

## Ethics statement

The studies involving humans were approved by According to the Declaration of Helsinki, the study protocol was approved by hospital ethics and research committee (registry number: HIM-2020-023). The studies were conducted in accordance with the local legislation and institutional requirements. Written informed consent for participation in this study was provided by the participants’ legal guardians/next of kin.

## Author contributions

IP-O: Conceptualization, Investigation, Writing – original draft. JZ-C: Conceptualization, Data curation, Formal analysis, Methodology, Writing – original draft. IO-F: Writing – review & editing. BR-N: Investigation, Writing – review & editing. MV-K: Supervision, Writing – original draft, Writing – review & editing. BM: Data curation, Investigation, Writing – review & editing. VD-C: Investigation, Writing – review & editing. JR-V: Data curation, Investigation, Writing – review & editing.
